# Bicaudal Is a Conserved Substrate for *Drosophila* and Mammalian Caspases and Is Essential for Cell Survival

**DOI:** 10.1371/journal.pone.0005055

**Published:** 2009-03-30

**Authors:** Emma M. Creagh, Gabriela Brumatti, Clare Sheridan, Patrick J. Duriez, Rebecca C. Taylor, Sean P. Cullen, Colin Adrain, Seamus J. Martin

**Affiliations:** Molecular Cell Biology Laboratory, Department of Genetics, The Smurfit Institute, Trinity College, Dublin, Ireland, United Kingdom; The Research Institute for Children at Children's Hospital New Orleans, United States of America

## Abstract

Members of the caspase family of cysteine proteases coordinate cell death through restricted proteolysis of diverse protein substrates and play a conserved role in apoptosis from nematodes to man. However, while numerous substrates for the mammalian cell death-associated caspases have now been described, few caspase substrates have been identified in other organisms. Here, we have utilized a proteomics-based approach to identify proteins that are cleaved by caspases during apoptosis in *Drosophila* D-Mel2 cells, a subline of the Schneider S2 cell line. This approach identified multiple novel substrates for the fly caspases and revealed that bicaudal/βNAC is a conserved substrate for *Drosophila* and mammalian caspases. RNAi-mediated silencing of bicaudal expression in *Drosophila* D-Mel2 cells resulted in a block to proliferation, followed by spontaneous apoptosis. Similarly, silencing of expression of the mammalian bicaudal homologue, βNAC, in HeLa, HEK293T, MCF-7 and MRC5 cells also resulted in spontaneous apoptosis. These data suggest that bicaudal/βNAC is essential for cell survival and is a conserved target of caspases from flies to man.

## Introduction

Members of the caspase family of cysteine proteases are involved in coordinating the terminal events of apoptosis in organisms as divergent as nematodes and mammals [1]–[4]. Caspases typically exist as dormant proenzymes in healthy cells and become activated at the onset of apoptosis through recruitment to activation scaffolds or membrane receptor complexes [5]–[8]. Upon activation, caspases target a subset of the proteome for restricted proteolysis and this results in a stereotypical series of events involving nuclear condensation and fragmentation, extensive plasma membrane blebbing, the appearance of ligands for phagocytic cells on the external leaflet of the plasma membrane, and in many cases the collapse of the cell into small fragments; presumably to facilitate removal of the dying cell [9].

While numerous substrates for human and mouse caspases have now been identified [9], [10], it is not clear whether the same subset of proteins is targeted for proteolysis by caspases in other organisms. For example, while the nematode worm *Caenorhabditis elegans* was instrumental in the initial discovery of a role for caspases in cell death control [11], [12], substrates for the major worm caspase, CED-3, have only recently been reported [13]. The fly has also proved to be a very instructive model for the study of the role of caspases and their regulators in developmental cell death [14]–[17], however, it remains unclear precisely how caspases coordinate apoptosis in this organism.

Because the phenotype of cells undergoing apoptosis in flies and mammals share remarkable similarities [18], it is likely that at least overlapping cohorts of caspase substrates undergo apoptosis-associated proteolysis in both phyla. To date, seven caspases have been identified in *Drosophila* and, of these, Dronc and DrICE appear to play particularly significant roles in the coordination of programmed cell death in this organism [19]. Dronc is the only CARD-carrying caspase in the fly and can associate with the adaptor molecule, Ark, to form a multi-subunit apoptosome complex in response to developmental triggers of apoptosis as well as toxic stimuli [20], [21]. Upon activation within the apoptosome, Dronc can promote activation of other caspases such as DrICE and DCP-1 [22], [23], thereby initiating a protease cascade. DrICE appears to represent the major effector caspase in the fly and may be the functional equivalent of mammalian caspase-3 in this organism [22].

At present, three substrates for *Drosophila* caspases, DIAP1, Lamin DmO, and the Drosophila Apaf-1 homologue, ARK, have been identified [22], [24], [25]. To gain further insight into how caspases contribute to programmed cell death in *Drosophila*, and to identify caspase substrates that are conserved between species, we have conducted a proteomics-based screen to search for proteins that undergo caspase-dependent proteolysis during apoptosis of *Drosophila* D-Mel2 cells. This analysis resulted in the identification of 14 proteins that underwent caspase-dependent alterations to their relative mobilities on two-dimensional (2D) SDS-PAGE gels. We have cloned two of these substrates and confirm that they are efficiently cleaved by the *Drosophila* caspases DrICE and DCP-1. We also show that the human homologue of one of these substrates, bicaudal, is also cleaved by caspases during apoptosis of mammalian cells. Bicaudal and its human homologue, βNAC, appear to be essential for cell proliferation and cell survival as RNAi-mediated silencing of the expression of these proteins resulted in a block to cell division, followed by spontaneous apoptosis. This suggests that, as well as targeting substrate proteins that contribute to the ordered destruction of the cell, caspases also inactivate key proteins such as βNAC that are essential for cell survival.

## Results

### Apoptosis in *Drosophila* D-Mel2 cells shares many features in common with apoptosis in mammalian cells and is caspase-dependent

**Figure 1:**
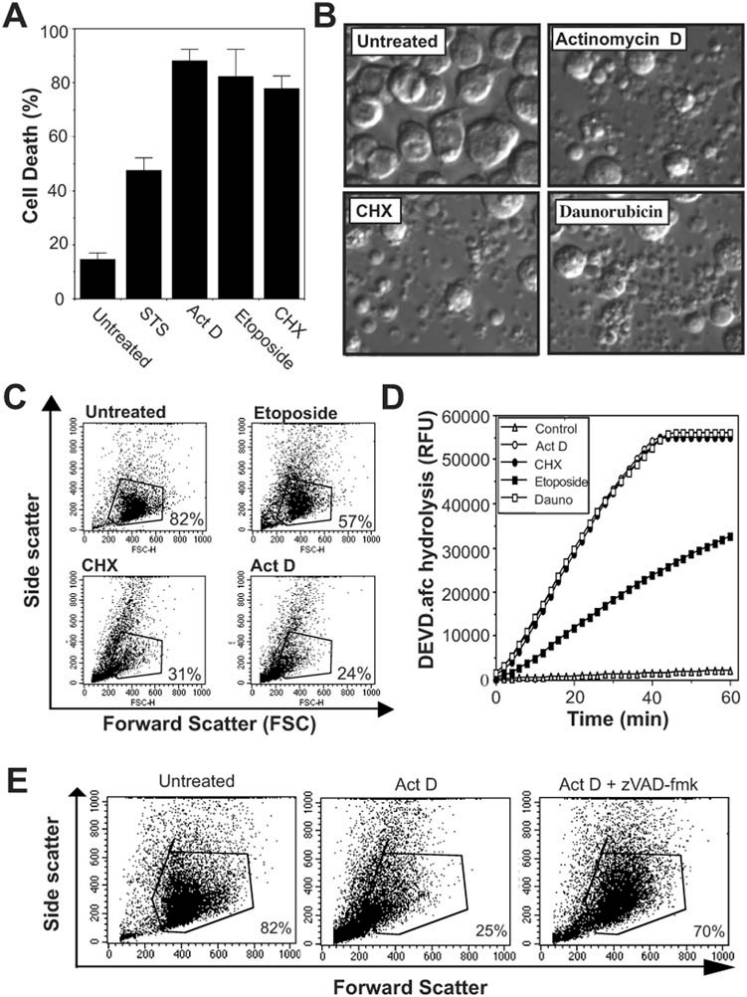
*Drosophila* cells undergo apoptosis in response to diverse stresses. (A) *Drosophila* D-Mel2 cells were treated with staurosporine (STS; 50 nM), actinomycin D (Act D; 600 nM), etoposide (10 µM) cycloheximide (CHX; 25 µM), or left untreated. The percentage of cells undergoing apoptosis in each culture was determined by direct morphological assessment after 18 h. (B) D-Mel2 cells were treated with actinomycin D (600 nM), cycloheximide (25 µM) daunorubicin, (20 µM), or left untreated, and phase contrast images were taken at 18 h. (C) D-Mel2 cells treated for 18 h with etoposide (10 µM), cycloheximide (25 µM) or actinomycin D (600 nM), or left untreated, were analysed by flow cytometry to determine the degree of cell death. The percentage of viable cells in each culture is indicated on each dot plot. (D) *Drosophila* D-Mel2 cells were treated either with actinomycin D (600 nM), cycloheximide (25 µM), etoposide (10 µM), or daunorubicin (20 µM), or left untreated. Cell lysates were prepared from treated cells after 18 h and levels of DEVD-AFC hydrolysis activity measured by the addition of lysate samples to reactions containing 50 µM DEVD-AFC, followed by measurement of the release of free AFC by fluorimetry. (E) *Drosophila* D-Mel2 cells were either left untreated, or were treated with actinomycin D (600 nM) in the presence or absence of z-VAD-fmk (20 µM). After 18 h, percentages of viable cells were measured by flow cytometry. Results are representative of three independent experiments.

To search for substrates for the fly caspases, we used a subline of the Schneider (S2) cell line, D-Mel2, that can be induced to undergo apoptosis in response to many of the same stimuli that promote apoptosis in mammalian cells. D-Mel2 cells exposed to staurosporine, actinomycin D, etoposide or cycloheximide died rapidly (Fig. 1A), and displayed typical apoptotic features such as cell shrinkage, extensive plasma membrane blebbing and cellular collapse into numerous apoptotic bodies (Fig. 1B and C). Robust caspase activation, as assessed by hydrolysis of the synthetic caspase substrate DEVD-AFC, was also readily detected in lysates generated from apoptotic D-Mel2 cells but not from control cell lysates (Fig. 1D). All of these events were caspase dependent and could be abolished with the poly-caspase inhibitor z-VAD-fmk (Fig. 1E and data not shown).

### Two-dimensional SDS-PAGE analysis of caspase-dependent changes to the D-Mel2 cell proteome during apoptosis

**Figure 2:**
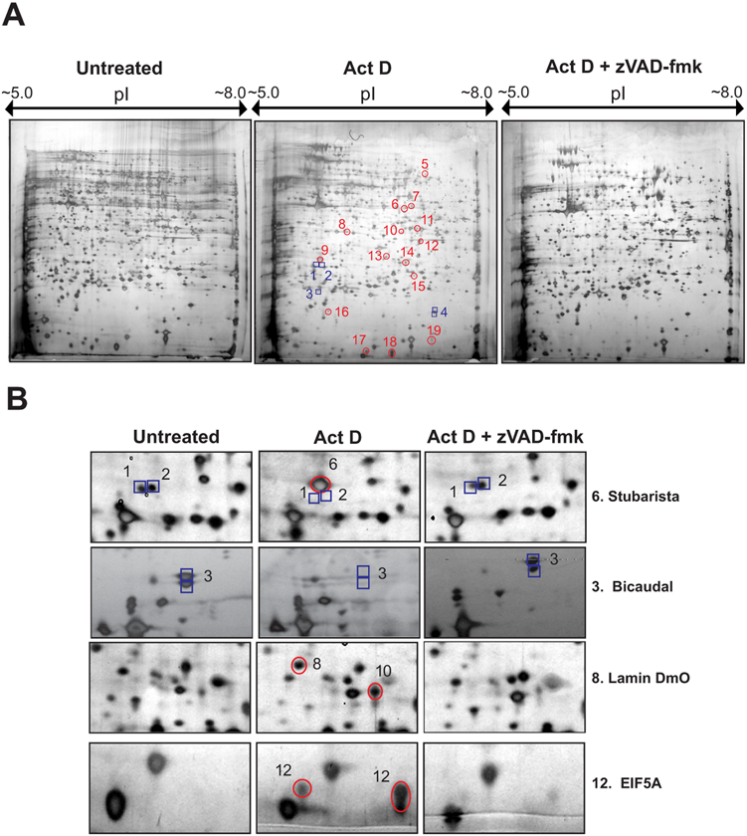
Detection of caspase-dependent alterations to the *Drosophila* proteome. (A) *Drosophila* D-Mel2 cells were either or left untreated, or were treated with actinomycin D (600 nM) in the presence or absence of z-VAD-fmk (20 µM). After 7 h, cell lysates were prepared and 500 µg of protein from each reaction was analysed by two-dimensional SDS-PAGE followed by silver-staining. Representative 2D gels are shown with caspase-induced alterations annotated. (B) Enlarged regions of silver-stained two-dimensional SDS-PAGE gels from control or actinomycin D-treated cell lysates are shown. Proteins reproducibly altered upon caspase activation are indicated. Protein identifications were obtained by MALDI-TOF mass spectrometry. Results are representative of at least three independent experiments. Blue squares represent protein spots that disappear, in a caspase-dependent manner, upon induction of apoptosis. Red circles represent new protein spots that appear in apoptotic cells. Spot numbers on the gel in Figure 2B refer to the protein numbers in Table 1.

To identify caspase-dependent alterations to the D-Mel2 proteome, we compared two-dimensional protein spot patterns from D-Mel2 cells induced to undergo apoptosis in the presence or absence of z-VAD-fmk (Fig. 2). Approximately 2,000 of the most abundant soluble proteins expressed in D-Mel2 cells could be resolved using 2D gels. We identified numerous alterations to the proteome of apoptotic cells, all of which were abolished in the presence of z-VAD-fmk suggesting that the observed changes were caspase-dependent protein modifications (Fig. 2).

Proteins that underwent caspase-dependent proteolysis during apoptosis of D-Mel2 cells were identified by MALDI-TOF mass spectrometry (Table 1). The 14 identified substrates include three chaperone proteins, three proteins involved in translation, and proteins involved in a variety of other functions. Two of the putative caspase substrates, bicaudal and stubarista, were chosen for more detailed analysis.

**Table 1:** Identities of *Drosophila* proteins that underwent caspase-dependent modification during apoptosis.

Spot	Protein ID	Accession no. (NCBI)	Human homologue	Peptide coverage	Peptide matches
**1**	Cu-chaperone for superoxide dismutase	gi 12963893	46% ID to Cu-chaperone for SOD	61%	14
**2**	CG-17753	gi 24652342	46% ID to Cu-chaperone for SOD	45%	10
**3**	Bicaudal	gi 24653216	57% ID to β-NAC	49%	8
**4**	CG-6428	gi 24639609	45% ID to hypothetical XP_375107	21%	10
**5**	Inorganic pyrophosphatase	gi 44888980	52% ID to inorganic PPase 2	48%	13
**6**	Stubarista	gi 12249039	73% ID to 40S ribosomal protein (P40)	60%	13
**7**	GH-18370	gi 16183135	50% ID to Tim44 mitochondrial translocase	36%	17
**8**	Lamin DmO	gi 1346410	33% ID to Lamin A	30%	21
**9**	CG-10992	gi 18921171	58% ID to Cathepsin B	30%	11
**10**	CG-11999	gi 24644197	59% ID to dihydropyrimidinase-like2	62%	21
**11**	Hsp23	gi 17737553	50% identity to Hsp27	47%	10
**12**	Eukaryotic translation factor 5A (eIF5a)	gi 10441423	69% ID to eIF5a, isoform 1	69%	11
**13**	CG-14996	gi 21355917	43% ID to neuronal protein NP25	60%	14
**14**	Heterogeneous nuclear ribonucleoprotein	gi 1346955	52% ID to DAZ-associated 1 isoform A	26%	8

### Bicaudal and stubarista are *bona fide* substrates for *Drosophila* caspases

**Figure 3:**
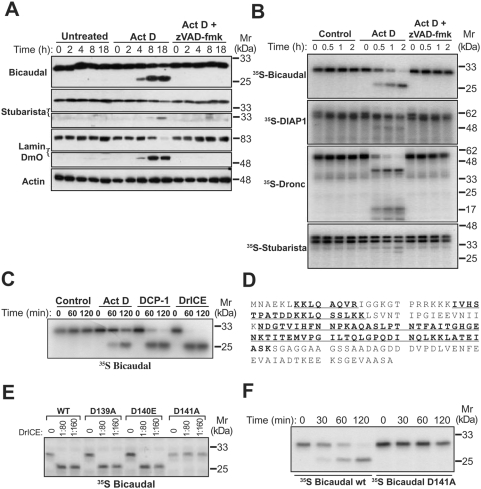
Bicaudal is cleaved by *Drosophila* caspases at residue D141. (A) *Drosophila* D-Mel2 cells were either left untreated, or were incubated with actinomycin D (600 nM) in the presence or absence of z-VAD-fmk (20 µM). At the indicated times, lysates were prepared and analysed by Western blotting with antibodies directed against substrate and control proteins. (B) Lysates prepared from D-Mel2 cells treated with actinomycin D (100 nM) for 18 h were incubated with ^35^S-methionine-labelled bicaudal, DIAP1, stubarista and Dronc. Samples were taken at the indicated times and analysed by SDS-PAGE/fluorography. (C) ^35^S-methionine-labeled bicaudal was incubated either with actinomycin D-treated D-Mel2 cell lysate as above, or with recombinant DCP-1 and drICE, and samples taken at the indicated times followed by SDS-PAGE/fluorography analysis. (D) Peptide coverage of the caspase-cleaved bicaudal protein sequence obtained by mass spectrometry is underlined. (E) Individual aspartic acid residues in the bicaudal sequence, D139, D140 and D141 were converted by site-directed mutagenesis to alanine (aa139 and aa141) or glutamic acid (aa140) residues. ^35^S-methionine-labelled wild type and mutant proteins were incubated in buffer or with recombinant drICE for 120 min, before analysis by SDS-PAGE and fluorography. (F) ^35^S-methionine-labeled wild type and D141A mutant bicaudal proteins were incubated in actinomycin D-treated cell lysate as above and samples taken at the indicated times for analysis by SDS-PAGE/fluorography.

To confirm that bicaudal and stubarista were genuine substrates for *Drosophila* caspases, we generated polyclonal antibodies against these proteins and used these to probe cell lysates prepared from healthy versus apoptotic D-Mel2 cells. As illustrated in Fig. 3A, bicaudal underwent rapid proteolysis upon induction of apoptosis and was cleaved with similar kinetics to lamin DmO. Proteolysis of stubarista was less efficient, relative to bicaudal and lamin DmO, being detected later and only a fraction of the total cellular complement of stubarista appeared to be cleaved under the conditions employed (Fig. 3A). Proteolysis of all of these substrates was completely inhibited by z-VAD-fmk, confirming that these proteolytic events were caspase-dependent (Fig. 3A). Similar results were also observed when *in vitro* transcribed and translated forms of bicaudal and stubarista were incubated in cell-free extracts generated from apoptotic versus untreated D-Mel2 cells (Fig. 3B).

### Bicaudal is cleaved at a single caspase cleavage motif at Asp141

Bicaudal is a homologue of human βNAC, involved in binding to nascent chains as they emerge from the ribosome, and mutations in both *Drosophila* and murine *βNAC* lead to embryonic lethality [26]–[28]. In addition, mutations in the *C. elegans* homologue of bicaudal, ICD-1, are associated with elevated levels of programmed cell death during embryonic development [29]. We therefore decided to explore this substrate in further detail. We mapped the caspase cleavage site within bicaudal by *in vitro* transcribing and translating the protein and incubating with recombinant DCP-1 and DrICE, both of which have been implicated as effector caspases in the fly [22]–[23]. As shown in Fig. 3C, both DCP-1 and DrICE generated a bicaudal fragment identical in size to the cleavage product seen when ^35^S-labeled bicaudal was incubated with lysates generated from apoptotic D-Mel2 cells. We then incubated recombinant bacterially-expressed bicaudal with DCP-1 and analysed the resulting cleavage products by mass spectrometry. The peptide coverage of the major fragment of bicaudal indicated that the caspase cleavage site lay within the C-terminus of the protein, within the last 46 amino acids (Fig. 3D). Using this information, we inspected the bicaudal protein sequence and mutated three candidate aspartic acids at positions 139, 140 and 141. As Fig. 3 E illustrates, only one of these mutants, D141A, fully resisted proteolysis by recombinant DrICE and similar results were found using DCP-1 (data not shown). To further confirm this as the site of proteolysis during apoptosis, we then incubated wild-type ^35^S-labeled bicaudal, alongside the D141A bicaudal mutant, in extracts generated from apoptotic D-Mel2 cells. This experiment confirmed D141 as the site cleaved by caspases during apoptosis within the motif ^138^GDDD^141^ (Fig. 3F). This is a somewhat unusual motif for executioner caspases, as these typically prefer a DXXD consensus. However, little information concerning optimal cleavage site motifs for *Drosophila* caspases is available for useful comparisons to be made.

### RNAi-mediated knockdown of bicaudal expression blocks proliferation and results in spontaneous apoptosis

**Figure 4:**
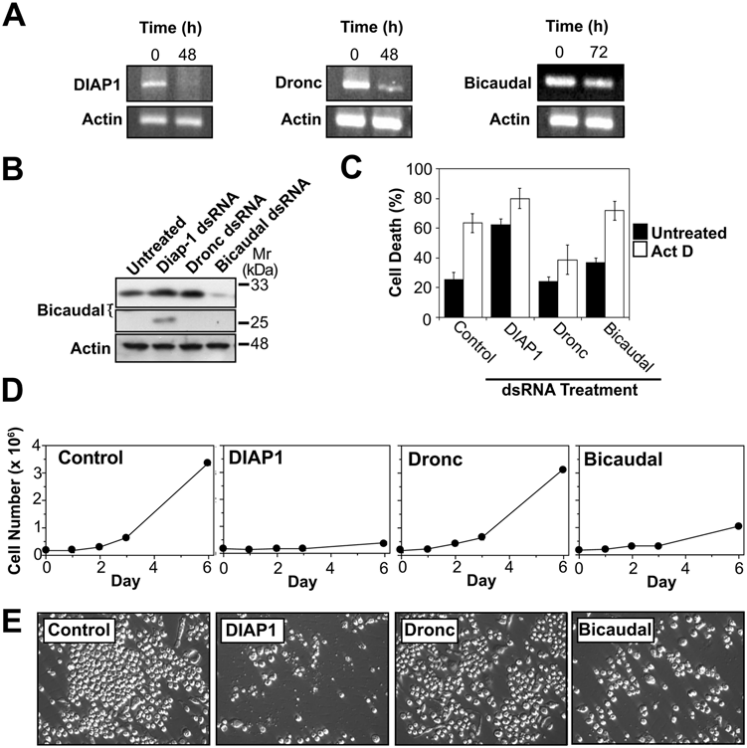
Reduced levels of bicaudal provokes spontaneous death of *Drosophila* cells. (A) *Drosophila* D-Mel2 cells were treated with dsRNAs against *diap1*, *dronc* or *bicaudal* for 48 or 72 h and levels of each mRNA was then determined by RT-PCR analysis. (B) D-Mel2 cells were treated with the indicated dsRNAs and lysates prepared after 48 h. Samples were subjected to SDS-PAGE and Western blotting with antibodies specific for bicaudal or actin. (C) D-Mel2 cells were treated with the indicated dsRNAs for 48 h followed by addition of actinomycin D (600 nM). Levels of cell death in drug-treated versus untreated cultures were then assessed. Results shown are representative of three independent experiments ±SEM. (D) D-Mel2 cells were treated with indicated dsRNAs and the number of cells present in each culture after 0, 1, 2, 3 and 6 days was assessed by haemocytometer counts. Results shown are the mean of triplicate counts ±SEM, from a representative experiment. (E) D-Mel2 cells were treated with dsRNAs targeted against the indicated gene products and phase contrast images of cultures were taken after 3 days. Results are representative of at least three independent experiments.

To explore the importance of Bicaudal for cell viability, we silenced expression of the *bic* transcript using double stranded RNA targeted against this gene. As controls, we also silenced expression of *dronc* and *diap1*, both of which are well-established regulators of programmed cell death in the fly. RNAi-mediated silencing of all of these genes was confirmed by RT-PCR analysis (Fig. 4A), and knockdown of bicaudal protein was also confirmed by Western blot analysis (Fig. 4B). Previous studies have shown that loss of *diap1* expression results in spontaneous apoptosis in the fly and in cell culture [30], [31], and we confirmed this observation (Fig. 4C and D). In contrast, silencing of *dronc* expression had no effect on cell viability or rates of proliferation (Fig. 4C and D), but did block apoptosis induced by exposure to cytotoxic drug treatment (Fig. 4C). However, *bic* knockdown resulted in a profound block to proliferation and cells also underwent spontaneous apoptosis but with slower kinetics to that seen due to loss of *diap1* (Fig. 4C–E). Furthermore, cells lacking bicaudal were also sensitized to apoptosis triggered by actinomycin D whereas knockdown of *dronc* expression protected from this stimulus, as expected (Fig. 4C). These data suggest that bicaudal expression is important for the sustained viability of *Drosophila* cells and that caspase-dependent proteolysis of bicaudal during apoptosis may contribute to cellular demise.

### βNAC, a human homologue of bicaudal, is a substrate for caspases and granzyme B

**Figure 5:**
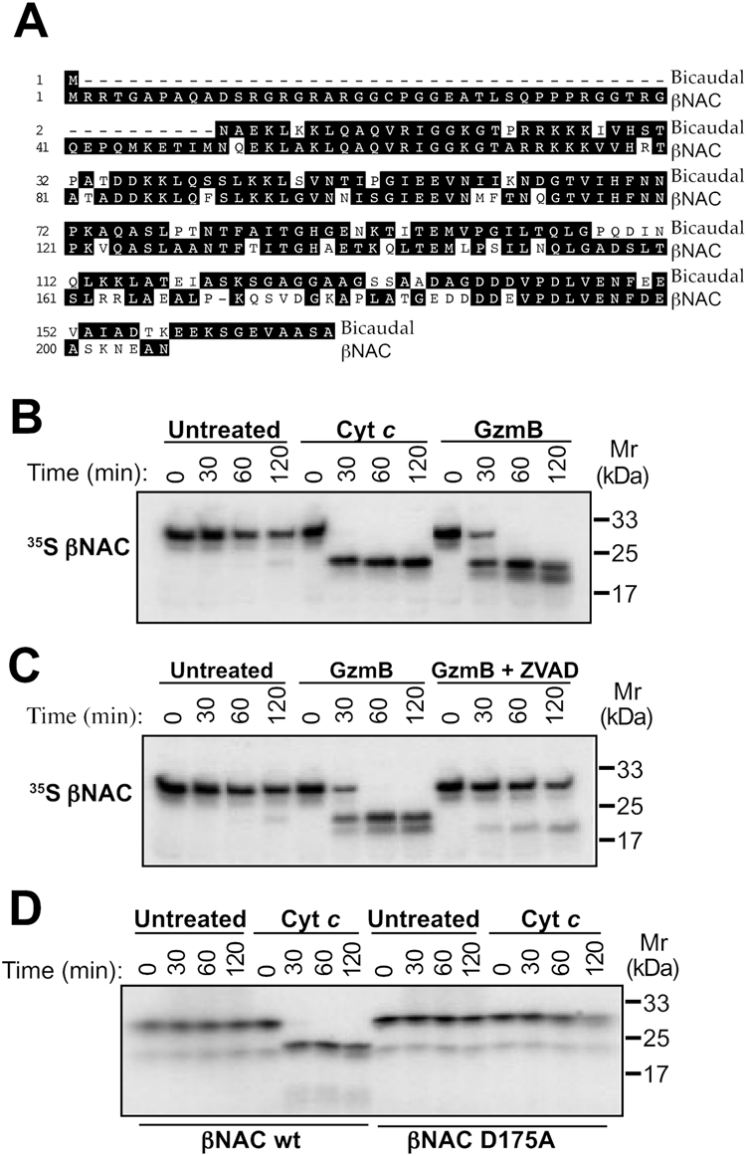
The human homologue of bicaudal, βNAC, is a substrate for human caspases and granzyme B. (A) Alignment of the *Drosophila* bicaudal and human βNAC amino acid sequences. (B) Human Jurkat cell-free extracts were treated with 50 µg/ml cytochrome c and 1 mM dATP, or 100 nM granzyme B, to activate caspases, or left untreated. ^35^S-methionine-labelled βNAC was added and samples taken after 0, 30, 60 and 120 minutes for analysis by SDS-PAGE and fluorography. (C) Human Jurkat cell-free extracts were treated with 100 nM granzyme B with or without 10 µM z-VAD-fmk, or left untreated. ^35^S-methionine-labelled βNAC was added and samples taken as above. (D) Residue D175 in βNAC was mutated to alanine by site-directed mutagenesis and ^35^S-methionine-labelled wild-type and mutant βNAC added to cytochrome c/dATP-treated or untreated cell-free extracts. Samples were then taken and analysed as above.

To further explore the significance of bicaudal for apoptosis, we then explored whether the human homologue of this protein, βNAC, the β-subunit of the nascent polypeptide-associated complex, was also cleaved by caspases during apoptosis. A previous study has indicated that βNAC may be cleaved in a caspase-dependent manner during apoptosis [32], however, the functional consequences of this was not examined. An alignment between bicaudal and βNAC revealed very extensive sequence identity between these two proteins of approximately 57% (Fig. 5A). We then explored whether βNAC underwent caspase-dependent proteolysis using a cell-free system based upon homogenates generated from human Jurkat cells [33]. Caspase activation in this system can be readily initiated either through introduction of the apoptosome co-factors, cytochrome c and dATP, or by introduction of the CTL/NK protease, granzyme B [34]. As, shown in Fig. 5B and C, βNAC was very efficiently cleaved upon activation of caspases by cytochrome c or granzyme B. Interestingly, granzyme B also produced an additional βNAC cleavage product, suggesting that the latter may also be a direct substrate for this granzyme. This was confirmed upon incubation of βNAC with granzyme B in the presence of z-VAD-fmk (to block caspase activity) where only the faster mobility βNAC cleavage product, seen only in the presence of granzyme B, was now observed (Fig. 5C).

### Mapping of the caspase cleavage site within βNAC

The aspartic acid within bicaudal that is targeted for cleavage by *Drosophila* caspases is altered to a glutamic acid at the equivalent position within human βNAC (Fig. 5A). Therefore we mutated alternative potential caspase cleavage motifs and found that βNAC is instead cleaved at Asp 175. Consistent with this, a D175A point mutant of βNAC completely resisted caspase-dependent proteolysis under conditions where the wild-type βNAC protein was completely cleaved by caspases (Fig. 5D).

### Silencing of βNAC expression results in proliferative arrest and spontaneous apoptosis of mammalian cells

**Figure 6:**
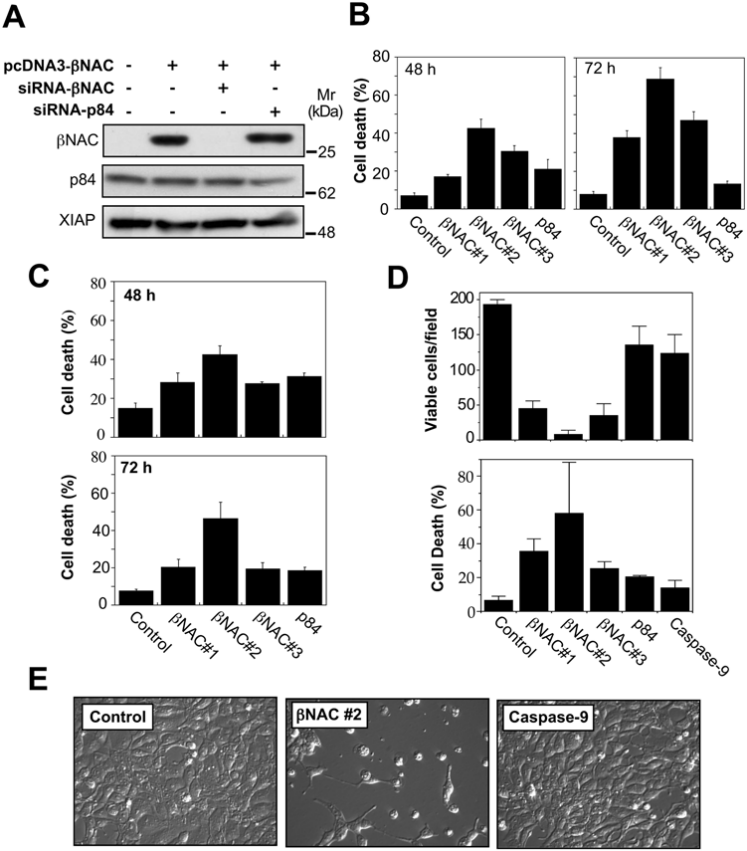
Reduction in βNAC protein levels results in a block to proliferation and spontaneous death in a range of human cell types. (A) MCF7 cells were transfected with pcDNA3-βNAC and treated with siRNA oligos against *βNAC*, or *p84* as a control. Lysates were prepared after 72 h and samples probed with antibodies against βNAC, p84 and XIAP. (B) MCF7 cells were treated with three siRNA oligos against *βNAC* and with an siRNA oligo against *p84*. After 48 and 72 h, the percentage of cell death in each culture was assessed. (C) MRC5 cells were treated with siRNA oligos and cell death levels assessed as described above. (D) HEK293T cells were treated with siRNA oligos against *βNAC*, *p84* and *casp-9* for 48 h and cell viability and cell death in each culture was assessed. (E) HEK293T cells were treated with siRNA oligos against *βNAC* or *CASP-9* for 48 h and phase contrast images taken.

To explore whether βNAC expression was also required for survival of mammalian cells, we silenced expression of this protein using siRNA directed against the *βNAC* coding sequence. To minimize the possibility of non-specific off-target effects, we designed three different siRNAs against the *βNAC* mRNA. As controls, we employed siRNAs targeted against *CASP-9* and *P84*, an irrelevant gene in this context. siRNA-mediated knockdown of βNAC expression was confirmed by Western blot analysis (Fig. 6A). As Fig. 6B illustrates, knockdown of *βNAC* expression in MCF7 cells resulted in a dramatic reduction in cell numbers over 72 h and a substantial increase in apoptosis during this time course. Essentially identical results were observed in untransformed MRC-5 human fibroblasts (Fig. 6C), HEK293T cells (Fig. 6D and E) and HeLa cells (data not shown). Collectively, these data indicated that βNAC serves an essential function in diverse cell types and proteolysis of this protein by caspases may contribute to the irreversible termination of cell viability during apoptosis.

## Discussion

Here we have identified several substrates for the *Drosophila* cell death-associated caspases. Previously, few substrates for caspases activated during cell death in the fly have been reported and the substrates reported herein provide a basis for further investigations exploring the functional significance of those proteolytic events. Furthermore, three of the substrates identified in this study, bicaudal, a heterogeneous nuclear ribonucleoprotein, and lamin DmO, are homologues of proteins known to be substrates of caspases in human cells [9], [10], [32]. Although it is commonly assumed that caspases promote the characteristic features of apoptosis by cleaving overlapping sets of substrates in divergent organisms, very few conserved caspase substrates have been identified to date.

One such substrate, the β-subunit of the nascent polypeptide-associated complex, bicaudal/βNAC, was efficiently cleaved by both *Drosophila* and human caspases. Interestingly, βNAC has also previously been implicated as a negative regulator of programmed cell death in the nematode *C. elegans* and ablation of this gene product results in massive and unscheduled apoptosis in developing worm embryos [29]. NAC functions to bind short nascent polypeptides as they emerge from the ribosome [26]. The latter event prevents inappropriate interactions with cellular proteins and non-specific binding by the signal recognition particle and consequent targeting to the ER [26], [35]. NAC also prevents the targeting of non-translating ribosomes to the ER [26], [35]. This fundamental role of NAC is reflected in the catastrophic phenotype of null mutations affecting the βNAC-coding sequence gene in a range of species. Loss of βNAC in developing mice leads to post-implantation lethality and mutation of *Drosophila* bicaudal promotes developmental arrest, which is associated with duplication of the posterior embryonic regions in the place of the anterior embryonic segments [27], [28]. As mentioned earlier, RNAi-mediated silencing of the *C. elegans* βNAC homologue, ICD-1, also results in developmental arrest associated with massive cell death [29]. Thus, the disablement of βNAC function through caspase-dependent proteolysis may contribute substantially to cellular demise.

In line with this, here we have shown that interference with βNAC expression levels in *Drosophila* or human cell lines leads to proliferative arrest, followed by cell death. However, although silencing of βNAC expression may replicate one possible consequence of caspase-dependent inactivation of this protein, it is also possible that caspase-mediated proteolysis of βNAC could generate a cleaved form of this protein with altered function (such as dominant-negative inhibition of native βNAC) that gene silencing may not faithfully reproduce. It is also important to note that expression of non-cleavable β-NAC in human cells did not give rise to any obvious change in the apoptotic phenotype (data not shown). This suggests that caspase-dependent proteolysis of β-NAC does not contribute to any obvious morphological feature of apoptosis. However, we propose that at least two functional categories of caspase substrates may be important in apoptosis. One category of caspase substrates may contribute to specific morphological features of this mode of cell death. Examples of such substrates include ICAD, the inactivation of which results in activation of the CAD endonuclease that contributes to apoptosis-associated DNA hydrolysis [36], [37]. Similarly, proteolysis of ROCK I is thought to result in the extensive plasma membrane blebbing and nuclear fragmentation that typifies apoptotic cells [38]–[40]. On the other hand, it is very likely that other proteins are cleaved by caspases in order to terminate the life of the cell, thereby contributing to the irreversibility of this process. We suggest that bicaudal/βNAC may represent a caspase substrate belonging to the latter category.

In summary, here we have provided the first snapshot of the proteins that become targeted for proteolysis by caspases during programmed cell death in a *Drosophila* cell line. Clearly, numerous additional substrates for the fly caspases are likely to exist but will require techniques that penetrate deeper into the fly proteome to identify these. However, our study provides direct evidence that caspases most likely coordinate apoptosis in divergent organisms through cleaving overlapping cohorts of cellular substrates.

## Materials and Methods

### Materials

Antibodies specific to *Drosophila* Lamin DmO and Actin were purchased from the Developmental Studies Hybridoma Bank (USA). Polyclonal antibodies to *Drosophila* Bicaudal and Stubarista were generated by repeated immunization of rabbits with recombinant polyhistidine-tagged forms of these proteins. Recombinant polyhistidine-tagged Bicaudal and Stubarista were expressed and purified from bacteria (data not shown). The peptides, z-VAD-fmk, Ac-DEVD-AFC, Ac-LEHD-AFC, and Ac-YVAD-AFC were all purchased from Bachem (UK). 17 cm IPG strips (pH 3–6 and pH 5–8), easymelt agarose and Bio-Lyte ampholytes™ were purchased from Biorad (UK). Unless otherwise indicated, all other reagents were purchased from Sigma (Ireland) Ltd.

### Cell culture

The S2 subline, D-Mel-2, are a subline of Schneider S2 insect cells isolated from late stage *Drosophilia melanogaster* embryos [41], and were cultured at 28°C/0.5% CO_2_ in serum-free medium (Invitrogen, UK). HeLa and MRC-5 cells were cultured at 37°C in RPMI 1640 containing 5% foetal calf serum and HEK293T cells were cultured in DMEM containing 10% foetal calf serum.

### Flow cytometry

To quantify levels of apoptosis-associated cell shrinkage and fragmentation, forward scatter (FSC) and side scatter (SSC) of cell populations were measured by flow cytometery (FACSCalibur, Becton Dickinson, CA).

### Preparation of cell-free extracts

Cell-free extracts of *Drosophila* D-Mel-2 cells were prepared by allowing cells to swell for 20 minutes on ice in CEB containing 250 mM sucrose (CEB: 20 mM HEPES-KOH, pH7.5, 10 mM Kcl, 1.5 mM MgCl_2_, 1 mM EDTA, 1 mM EGTA, 1 mM DTT, 100 µM PMSF, 10 µg/ml leupeptin, 2 µg/ml aprotonin). Cells were then lysed by homogenization with ∼20–30 strokes of a B-type pestle and crude extracts centrifuged for 15 mins at 15,000 g to remove nuclei, unbroken cells and other debris. Extracts from human cells were prepared as described previously [33].

### Two-dimensional (2D) gel electrophoresis

For 2D gel electrophoresis, cells were lysed in 2D sample buffer (8M Urea, 4% CHAPS, 100 mM DTT, 0.05% SDS, 0.5% ampholyte 3–10 and a trace of bromophenol blue) and were rehydrated into 17cm IPG strips (BioRad). Passive sample rehydration into IPG strips was performed at room temperature overnight. Isoelectric point focussing (IEF) was performed in a BioRad Protean IEF Cell under the following conditions: (1) linear voltage ramp to 500 V over 1 hr, (2) 5 hr at 500 V, (3) linear voltage ramp to 3500V over 5 hr and (4) 12 hr at 3500 V. Following IEF, the IPG strips were reduced and alkylated with 2% DTT and 2.5% IAA, respectively, in 5 min incubations in an equilibration buffer containing 6M Urea, 375 mM Tris HCl, pH 8.8, 2% SDS and 20% Glycerol. Strips were then mounted on 12% SDS-PAGE gels using easymelt agarose (Biorad, UK) and electrophoresed at 37.5 mA per gel in a Biorad Protean IIxi electrophoresis cell (Biorad, UK). 2D gels were either Coomassie-blue stained or stained using a Mass Spectrometry-compatible silver staining protocol that is based on a modification of the EMBL silver staining protocol [42].

### In-gel protein digestion and protein identification by MALDI-TOF mass-spectrometry

Protein spots were manually excised from 2D gels. Gel pieces were incubated at room temperature on a shaking platform in oxidation buffer (15 mM K_3_Fe(CN)_6_, 50 mM Na_2_S_2_O_3_) until the spots were completely destained. Gel pieces were then washed 5 times (5–10 min per wash) in 50% methanol/10% acetic acid. Samples were then incubated in 50 mM NH_4_HCO_3_ for 5 minutes, prior to dehydrating in 100% acetonitrile. To further dehydrate the pellets, the acetonitrile was aspirated off and samples were spun in a speed-vac (ThermoSavant) for 5 minutes at room temperature. For trypsin digestion, a 100 µg/ml aliquot of Sequencing grade trypsin (Roche) dissolved in 1 mM HCl was diluted 1∶10 in digestion buffer (25 mM NH_4_HCO_3,_ 0.1 n-octyl β-D-Glucopyranoside). Typically, for low abundance silver stained spots, 2 µl of trypsin solution (20 ng) was pipetted directly unto the desiccated gel piece. After allowing the gel piece to rehydrate for 5 minutes, a further 10 µl of digestion buffer was added and samples were incubated overnight at 37°C. Following trypsin digestion, peptides were extracted twice into 40 µl 66% acetonitrile/0.1% trifluoroacetic acid in a sonicating water bath, followed by lyophilization in a speed-vac at room temperature.

For mass-spectrometric analysis, peptides were solubilized by sonication in 5 µl of 5% formic acid. Digested samples (0.5 to 1 µl) were applied to a Teflon-coated 96-well MALDI target plate (Applied Biosciences, UK), followed by the addition of 0.5 to 1 µl of a 10 mg/ml matrix solution of α-cyano-4-hydroxy-cinnamic acid in 60% acetonitrile/0.1% trifluoroacetic acid. Samples were allowed to air-dry at room temperature before analysis in positive reflectron mode in a Voyager DE Pro MALDI mass spectrometer (Applied Biosciences, UK).

### Plasmids and site-directed mutagenesis

Site-directed mutagenesis was carried out using the Quickchange kit (Stratagene). All plasmids were verified by DNA sequencing.

### Expression and purification of *Drosophila* caspases

DCP-1 and DrICE were expressed in BL21 cells as polyhistidine-tagged fusion proteins and were purified over Nickel-NTA agarose (Qiagen, UK) followed by elution with 100 mM imidazole. Both proteases demonstrated robust proteolysis of the synthetic peptide substrate DEVD-AFC and their activities were normalized using this peptide.

### Fluorimetry assays

Reactions containing recombinant caspases or *Drosophila* cell-free extract were typically assembled in a final volume of 100 µl. Following incubation at 37°C for 15 min, 2.5 µl samples were diluted to a final volume of 200 µl in WCEB containing 50 µM Ac-DEVD-AFC. Samples were then measured in an automated fluorimeter (Spectrafluor Plus, TECAN, UK) at wavelengths of 430 nm (excitation) and 535 nm (emission).

### Coupled in vitro transcription and translation

^35^S-methionine-labelled proteins were generated using the TNT kit (Promega) as described previously [33].

### RNAi and siRNA-mediated silencing of gene expression

RNAi-mediated silencing of gene expression in *Drosophila* was achieved using double-stranded RNA transcripts generated from the coding sequences of *diap-1*, *bicaudal* and *dronc*. All dsRNAs were generated using the Megascript kit (Ambion, UK) followed by purification of RNA using ethanol precipitation. For gene-silencing, ∼15 µg of dsRNAs were transfected into D-Mel2 cells followed by incubation for 72 h. For siRNA-mediated gene silencing, 21 base long oligonucleotides were generated to match the target sequence and were transfected into mammalian cells using oligofectamine (Invitrogen, UK). siRNA oligos were transfected at 200 nM, followed by incubation for 48–96 h.

### RNA analysis

To confirm knockdown following transfection with dsRNA, RNA was extracted from cells by lysis in RNeasy lysis buffer (RTL Buffer, Qiagen) with 1% β-mercaptoethanol, followed by addition of an equal volume of 70% ethanol. Lysates were applied to RNeasy columns (Qiagen) and eluted with ddH_2_O. Specific RNAs were then reverse-transcribed to cDNAs using the Omniscript kit (Qiagen) and analysed by electrophoresis through agarose gels.

### Image acquisition and analysis

The images presented in Fig. 1B, Fig. 4E and Fig. 6E were taken on an inverted microscope (IX71; Olympus) using a 40× objective, and images were captured using a Colorview II camera (Soft Imaging System) equipped with Analysis image acquisition software. All images were acquired at room temperature. All two-dimensional gel images were captured using a digital camera (CoolPix 5000; Nikon) and were imported into Photoshop (Adobe) for uniform brightness and contrast adjustment. All Western blot and fluorogram images were scanned from x-ray film using a scanner (Epson Perfection) and imported into Photoshop for uniform brightness and contrast adjustment.
