# Correction: Bicaudal Is a Conserved Substrate for *Drosophila* and Mammalian Caspases and Is Essential for Cell Survival

**DOI:** 10.1371/annotation/dbafbfb3-0c37-4e20-931a-8f041ff5d721

**Published:** 2009-03-31

**Authors:** Emma M. Creagh, Gabriela Brumatti, Clare Sheridan, Patrick J. Duriez, Rebecca C. Taylor, Sean P. Cullen, Colin Adrain, Seamus J. Martin

The author affiliation is incorrect. The correct affiliation is: Molecular Cell Biology Laboratory, Department of Genetics, The Smurfit Institute, Trinity College, Dublin, Ireland.

